# Wheat Escapes Low Light Stress by Altering Pollination Types

**DOI:** 10.3389/fpls.2022.924565

**Published:** 2022-06-09

**Authors:** Hong Yang, Yongpeng Li, Dongxiao Li, Liantao Liu, Yunzhou Qiao, Hongyong Sun, Wenwen Liu, Wenjun Qiao, Yuzhao Ma, Mengyu Liu, Cundong Li, Baodi Dong

**Affiliations:** ^1^Key Laboratory of Agricultural Water Resources, Hebei Laboratory of Agricultural Water-Saving, State Key Laboratory of North China Crop Improvement and Regulation, Center for Agricultural Resources Research, Institute of Genetics and Developmental Biology, Chinese Academy of Sciences, Shijiazhuang, China; ^2^State Key Laboratory of North China Crop Improvement and Regulation, College of Agronomy, Hebei Agricultural University, Baoding, China; ^3^College of Advanced Agricultural Sciences, University of Chinese Academy of Sciences, Beijing, China

**Keywords:** wheat, floret, grain number, low light stress, pollination way

## Abstract

Although low light stress seriously affects florets fertility and grain number during the reproductive period, crops can be fertilized by heterologous pollen to alleviate the reduction of grain number. However, wheat is strongly autogamous, how to change to outcross after low light remains unclear. To understand the mechanisms of this change process, an approach combined morphological, physiological, and transcriptomic analyses was performed under low light stress imposed at the young microspore stage the booting stage from tetrad to uni-nucleate microspores stage. The results showed that low light stress caused pollen abortion, and the unfertilized ovary is fertilized by heterologous pollen after floret opening. Compared to control, the opening angle of lemma and glume were increased by 11.6–48.6 and 48.4–78.5%, respectively. The outcross of stressed wheat compensated for the 2.1–18.0% of grain number loss. During this process, phytohormones played an important role. Jasmonic acid (JA) and methyl jasmonate (MeJA) levels in spikelets were increased. Meanwhile, lignin and cellulose content decreased, and genes associated with cell wall related GO terms were enriched. Among the differentially expressed genes (DEGs), were identified 88-710 transcription factors genes, of which some homologs in Arabidopsis are proposed to function in lignin and cellulose, influencing the glume and lemma opening. Our finding can provide new insight into a survival mechanism to set seeds through pollination way alteration in the absence of self-fertilization after the stress of adversity.

## Introduction

Wheat (*Triticum aestivum* L.) is the third largest food crop for approximately 60% of the world’s population (Food and Agriculture Organization [FAO], 2013). By 2050, wheat production will need to be doubled to meet the demands of the world’s growing population (Foulkes et al., 2011). Generally, crop production depends on a moderate natural environment for successful reproductive development (Wang et al., 2015). Unfortunately, extreme climate events, such as global dimming (reduction of global radiation), cold or drought, have occurred frequently in the past decades (Cardona et al., 2012), which limited crop growth and caused severe losses in grain yield (Ji et al., 2011). For instance, from 1960 to 2000, solar radiation at the global surface decreased by 1.3% per decade (Solomon et al., 2007). In China, solar radiation decreased by 5–30% over these years in some major wheat producing areas (Chameides et al., 1999), resulting in a 5.2% reduction in annual wheat production potential (Chen et al., 2013).

Grain number is one of the main components of yield. Stresses that happened during reproductive stages have the greatest effect on the formation of grain number, since it could dramatically induce floret sterility (Fischer, 2011). In self-fertilizing cereals, the successful development of pollen and ovary is essential for grain production, and abiotic stresses which interfere with the early development of pollen formation and ovaries can result in substantial grain loss (Jain et al., 2007; Endo et al., 2009). It has been shown that although low light stress leads to failure of florets (Dong et al., 2019), wheat produces seeds by capturing heterologous pollen. To overcome yield loss due to pollen failure caused by stresses, capturing heterologous mature pollen is one of the few strategies for yield stability under an adverse environment. Therefore, it is important to understand the mechanism of transition from self-fertilization to heterologous pollen fertilization of wheat as it links directly to ensure food security in extreme climates.

Wheat is a strict self-pollinating crop, with less than 1% probability of cross-pollination (Griffin, 1987; Martin, 1990; Hucl, 1996), which is due to its cleistogamic (closed) floral morphologies (Nguyen et al., 2015). Wheat florets are hermaphrodites. The small anthers and ovary are located at the base of the floret, surrounded by the palea and lemma, and several florets grew on one spikelet with two stiff and large glumes outside. This structure is suited for selfing. Normally at anthesis, the lemma and palea are pushed apart temporally (lasting for 8–30 min) by lodicules swelling, and the pollen dehiscences from anthers and falls on the ovary to accomplish fertilization (de Vries, 1971). However, some studies have found that wheat glumes and lemmas could open to receive heterologous pollen when the ovary is not pollinated within a few days post-anthesis (Kirby et al., 1983; Pickett, 1993). Moreover, wheat’s outcrossing rate is related to the degree of glume and lemma opening (Hucl, 1996), and the exsertion of stigmas (Xie et al., 2003).

It is known that the glume opening (first opening) in cereals is controlled by the swelling and subsequent withering of lodicules (Wang et al., 1988; Heslop-Harrison and Heslop-Harrison, 1996). This was mainly related to the weakening of the cell wall (Wang and Gu, 2012; Christiaens et al., 2016). Meanwhile, jasmonic acid (JA) and methyl jasmonate (MeJA) have been reported to influence the opening of glume (Yang J. et al., 2020). Okada et al. (2018) suggested that floret opening after anthesis (second opening) was related to the ovary size but not to the lodicules. The unfertilized ovary increases in radial dimension and produces lateral thrust of the rigid lemma and palea. Some studies are shown that the floret opening was mainly related to the uneven thickness of the cell wall in many cases of flowering plants (Ostergaard, 2009; Llorens et al., 2016; Zajczkowska et al., 2021). The cell wall composition and glume toughness was related to cellulose and lignin biosynthesis (Zou et al., 2015). Fu et al. (2017) found that carbohydrate metabolism, including the degradation of polysaccharides (such as cellulose and lignin) in cell walls, may play an important role in regulation of glume-unclosing after anthesis. However, it is still not clear whether the lemma and glume undergo deformations which could increase the efficiency of outcrossing during the “second opening”. Overall, the physiological and biological mechanisms of wheat glume opening have so far been poorly studied.

Extreme weather has become an increasing threat to wheat production. Thus, an intensive understanding of the mechanism of how wheat avoids or resists these stresses is important to reduce crop yield losses under adverse conditions. In this study, we used an integrated strategy combining transcriptome sequencing and morphophysiological analyses of the wheat spike at two stages (under low light stress period and stress removal until early grain filling stage) with following aims to evaluate the compensation effects of cross-pollination after low light stress; and to determine the mechanisms of how wheat switched the pollinating ways to avoid the low light stress. Our results provide insights into the mechanism of glume opening for cross-pollination after low light stress in this important crop.

## Materials and Methods

### Wheat Cultivars, Growing Conditions, and Stress Treatments

Two cultivars of winter wheat, namely ‘Henong825’ (‘HN825’) and ‘Kenong9204’ (‘KN9204’) were used, both of them were widely planted in North China Plain and were identified with different degrees of shade tolerance by our previous study (Dong et al., 2019).

**FIGURE 1:**
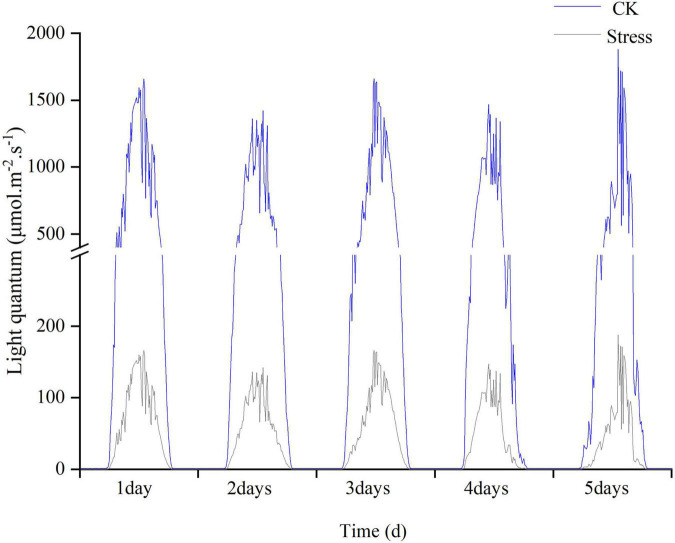
The light intensity under control and stress conditions was monitored during the experimental period in 2017–2018.

The field experiments were conducted at the Luancheng Agro-Ecosystem Experimental Station of Chinese Academy of Sciences (37°53′N, 114°41′E, altitude at 50 m), Hebei Province, China during the 2017/2018 and 2018/2019 winter wheat growing seasons. The soil was loam containing 21.41 g kg^–1^ organic matter, 109.55 mg kg^–1^ alkaline nitrogen, 1.44 g kg^–1^ total N, 15.58 mg kg^–1^ available phosphorus, and 220 mg kg^–1^ rapidly available potassium in the topsoil (0–40 cm) of the experimental plots. The study area is in a monsoon climate zone. In a previous manuscript (Yang H. et al., 2020), we have shown the mean temperature, total precipitation, and solar radiation in the winter wheat-growing season. The experiment was a randomized block design with three plot replicates. Each plot size was 6 m long and 2 m wide, with 40 rows. The experiment contained a low light treatment and control (CK). The low light treatment was performed as follows: 90% shading stress treatment was applied for 5 days at the YM stage (young microspore). YM stage was determined using the auricle distance (AD, the distance between the auricle of the flag leaf and the auricle of the penultimate leaf) of the main stem. The cultivar ‘HN825’ reached the YM stage at 1–2 cm, whereas ‘KN9204’ reached the YM stage at -1-0 cm (Dong et al., 2019). Shading stress treatment was applied by placing black polyethylene screens horizontally at the height of 2 m aboveground, reducing incident radiation (measured by a portable weather station, ECA-YW0501; Beijing, China) by 90 ± 5% during treatment imposition (Figure 1).

### Testing the Cross-Pollination Rate After Low Light Stress

After low light stress, randomly selected 60 main tillers were labeled, and 30 spikes from these main tillers were covered with white paper bags. At maturity, these spikes were harvested and threshed to determine the grain number in uncased and bagged spikes after low light stress. In addition, the compensation rate of cross-pollination after low light stress was the percentage of the yield difference between uncased and bagged spike to the yield difference between control and bagged spike:


$$\mathrm{Compensation}\,\mathrm{rate}\,\mathrm{of}\,\mathrm{cross}-\mathrm{pollination}=\frac{\left(\mathrm{Grain}\,\mathrm{number}\,\mathrm{of}\,\mathrm{uncased}\,\mathrm{spike}-\mathrm{Grain}\,\mathrm{number}\,\mathrm{of}\,\mathrm{bagged}\,\mathrm{spike}\right)\,}{\left(\mathrm{Grain}\,\mathrm{number}\,\mathrm{of}\,\mathrm{control}\,\mathrm{spike}-\mathrm{Grain}\,\mathrm{number}\,\mathrm{of}\,\mathrm{control}\,\mathrm{of}\,\mathrm{bagged}\,\mathrm{spike}\right)}$$

### Testing Anthers and Ovaries Fertility

To test the effect of low light stress on anthers and ovary fertility, stressed wheat and control wheat was used in cross-pollination experiments. Selected 20 plants were labeled from each of the stressed and control spikes in two cultivars. Ears of two wheat cultivars were covered with paper bags at heading stage to prevent uncontrolled cross-pollination. Crosses were conducted using control pollen a few days later when control ears were fully exerted and most of the florets were open. At maturity, grain number was determined and analyzed using *t*-tests.

### *In situ* Starch Localization and the Activity of Pollen Grains Determining

Fresh florets from control and stressed wheat plants were dissected from the ear. Anthers and ovaries staining were performed using I_2_-KI solution (0.20% w/w I_2_ and 0.50% w/w KI). The staining solution was allowed to penetrate the cells for 1 min at room temperature. After washing with deionized water, stained florets were viewed with a stereomicroscope.

For pollen staining, four florets in the middle of spike were harvested from each plant, and pollen was released by gentle pressure in 50 μL NanoPure water. I_2_-KI staining was added to the pollen, which was then incubated for 1 min at room temperature. Five individual plants from each treatment group were analyzed and viewed using a Leica DM6 microscope (Leica Microsystems, Wetzlar, Germany). Moreover, the viable pollen cells rate was measured using a pollen vitality analyzer (Ampha Z32, Switzerland).

To study young microspore development under low light stress, 20 anthers from each treatment group were harvested and then fixed using FAA (Formaldehyde-acetic acid-ethanol) stationary liquid. After 3 days, fixed anther samples were washed with 70% ethanol and stored at 4°C. The anthers were stained with 0.5 μg mL^–1^ DAPI [2-(4-Amidinophenyl)-6-indolecarbamidine dihydrochloride] to detect their nuclear development and pollen fertility, and photographs were taken using a Leica DM6 microscope.

### Measurements of Glume and Lemma Opening Angle

In the early grain filling stage (about 20 days after removing low light, which is the period of floret opening), ten spikelets from five labeled plants in each treatment were collected and taken images for glume and lemma opening angle measurement. The opening angle was analyzed by ImageJ software. Glume angle was measured between the tips of both glume and the connection point to the spikelet pedicel. Similarly, the lemma angle was measured between the tips of both lemma and the connection point to the spikelet pedicel.

### Measurements of Plant Endogenous Hormones

On the fifth day of low light stress and early grain filling stage (20 days after removing low light), spike tissues in each treatment were harvested and immediately frozen in liquid nitrogen and then stored at −80°C for subsequent measurement of hormones content. Jasmonic acid (JA) and methyl jasmonate (MeJA) was extracted at 4°C and three technical replicates were performed for each biological replicate. Measurement of JA and MeJA levels was carried out at Servicebio (Wuhan, China) using the LC-MS system. Three biological replicates in each treatment were measured.

### Lignin, Cellulose, and Hemicellulose Content Assay

Measurements of lignin content in the glume and lemma were performed with the acetylation method using a Plant Lignin assay kit (Suzhou Grace Biotech Co. Ltd., Suzhou, China) according to the manufacturer’s protocol. For each treatment group, three replicates were harvested on the fifth day of low light stress and early grain filling stage. Measurements of cellulose in the glume were performed with the reaction method of anthrone reagent with furfural compounds using a Plant Cellulose assay kit (Suzhou Grace Biotech Co. Ltd., Suzhou, China) according to the manufacturer’s protocol. Measurements of hemicellulose in the glume were performed using a Plant Hemicellulose assay kit (Suzhou Grace Biotech Co. Ltd., Suzhou, China) according to the manufacturer’s protocol.

### RNA Isolation, cDNA Construction, and Sequencing

Spikes of two cultivars were harvested on the fifth day of low light stress and early grain filling stage, respectively. Two biological replicate samples were collected for RNA-seq. Total RNA was extracted using Plant RNA Purification Reagent (Invitrogen, Carlsbad, CA, United States). The cDNA libraries were constructed using a Truseq™ RNA sample prep Kit (Illumina, San Diego, CA, United States) according to the manufacturer’s instructions. RNA sequencing was performed by Shanghai Majorbio Bio-pharm Technology Company using an Illumina NovaSeq 6000.

After sequencing, the clean reads were mapped to the *Triticum aestivum* genome (IWGSC, Ensembl Plants) by HISAT2. The FPKM (Fragments Per Kilobase of transcript per Million mapped reads) was calculated by RSEM, and DEGs (deferentially expressed genes) were identified using DESeq2. Genes with at least a two-fold change in expression level with a *P*-adjust value less than 0.05 were considered DEGs. Gene Ontology (GO) enrichment analysis was performed using ClusterProfiler3.0.

The transcription factor family analysis was performed as described previously (Li et al., 2018). The homologous genes in arabidopsis of DEGs were gained from Ensembl Biomart.

The scatter plots of GO enrichment results were drawn using ggplot2 in R packages, the heatmap of genes was visualized using Cluster3.0 and TreeView. Co-expression interactions of hub genes and the co-expressed genes were visualized using Cytoscape.

### Statistical Analysis

All data were analyzed using the SPSS 22.0 package. One-way ANOVA was used to determine the statistical significance between control treatment and shading stress for glume and lemma angle, and compensation rate, at *P* < 0.05 using the LSD value. The statistical significance of grain number, hormone content, lignin, cellulose, and hemicellulose content measurements was analyzed with a *t*-test. Difference was tested at *P < 0.05; **P < 0.01; ***P < 0.001.

## Results

### Low Light Changed the Fertilization Way of Wheat

**FIGURE 2:**
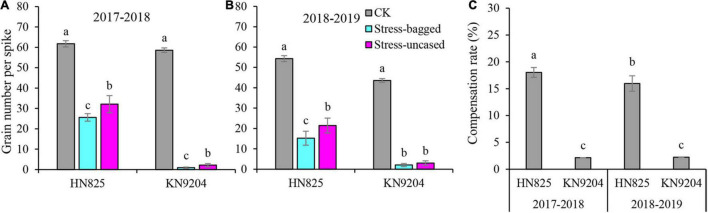
Testing the seed setting rate of heterologous pollen pollination of spike after low light stress in 2017–2018 **(A)** and in 2018–2019 **(B)**. **(C)** Compensation rate of heterologous pollen pollination of the spike to grain number after low light stress. CK: grain number per spike of control plants were grown under normal light conditions; Stress-bagged: grain number per spike with white bags after removing low light stress; Stress-uncased: grain number per spike without white bags after removing low light stress; Different letters in each cultivar indicate the statistically significant differences between treatments (*P* < 0.05) **(A,B)**.

In previous study, we found that low light stress can dramatically cause floret abortion, resulting in the reduction of grain number (Dong et al., 2019). The fact that pollen is more sensitive to adverse environments and that aborted pollen can induce ovary to receive heterogenous pollens (Chen et al., 2020). This reminded us that the switch of pollination way might exist in wheat under low light stress and could use to compensate for the yield loss. To verify it, we systematically investigated the grain number production of bagged spikes (performed after removing low light stress) and uncased spikes (Figures 2A,B). Compared with the bagged spike, the grain number of the uncased spike was significantly higher in both cultivars after low light stress treatment. The grain number of an uncased spike was 25.4–137.0% higher than that of bagged spike. The results suggested that low light stress can increase cross-pollination which would alleviate wheat grain number loss induced by low light. We then evaluated the compensation effect of cross-pollination on grain number per spike. The cross-pollination compensated 2.1–18.0% reduction of grain number per spike caused by self-pollination failure after low light stress (Figure 2C).

### Anthers Were More Sensitive to Low Light Stress Than Ovaries

**FIGURE 3:**
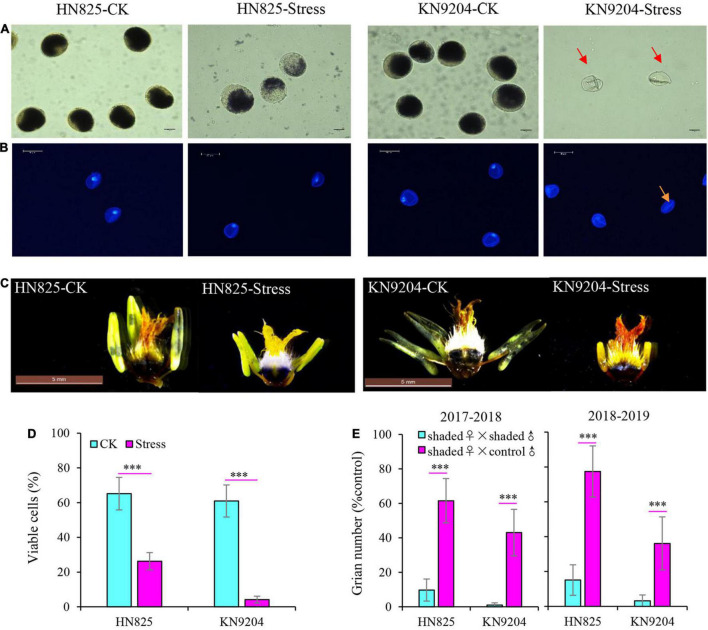
Effects of low light stress on the anther and ovary fertility in ‘HN825’ and ‘KN9204’. Pollen of the two cultivars was stained with I_2_-KI solution **(A)** and DAPI solution **(B)** to determine fertility and development. Starch staining was carried out on anthers and ovaries of control (CK) and stressed plants **(C)**. The rate of viable pollen cells in control and stress treatments were measured at anthesis **(D)**. Stressed wheat and control wheat was used in cross-pollination experiments to test the effect of low light stress on anthers and ovary fertility **(E)**. The scale bar indicates 20 μm **(A)**, 50 μm **(B)**, and 5 mm **(C)**, respectively. Shaded ♀ × shaded ♂ indicates that stressed ovary was pollinated with stressed pollens; Shaded ♀ × control ♂ indicates that stressed ovary was pollinated with control pollens **(D)**. Significant differences between treatments are indicated by asterisks (***P < 0.001, Student’s *t*-test).

The change of grain number is closely related to florets fertility. To determine floret fertility, anthers and ovaries were stained with I_2_-KI staining solution, and pollen grain development was determined by DAPI staining (Figure 3). For anthers, the fertility of pollen was significantly decreased under low light stress in both cultivars, and that of ‘KN9204’ was even completely sterile (Figure 3A). Moreover, the development of pollen in ‘KN9204’ was suppressed with the pollen nuclei disappearing under low light stress (Figure 3B). In addition, compared with control, the anther morphology of both cultivars was seriously abnormal, and the anther of ‘KN9204’ was sterile and shriveled (Figure 3C). Moreover, viable pollen cells rate was only 26.3 and 4.1% in ‘HN825’ and ‘KN9204’ after low light stress (Figure 3D). To further determine the effect of low light stress during the YM stage on wheat anther and ovary fertility, the cross-pollination experiments between control male fertile and stress female were performed (Figure 3E). The grain number of the self-pollination (the stressed ovary was pollinated with the fresh pollens from the stressed plants) is significantly lower than that of the cross-pollinated plants (the stressed ovary was pollinated with the fresh pollens from the control plants) in both cultivars. The grain number of self-pollination plant was only 1.0–15.1% of that of control, while the grain number of the cross-pollinated plant was 36.1–77.4%. This result suggested that the harmful effect of low light stress on anther was greater than that of the ovary, and the ovary was still fertile after 5 days of low light stress.

### Glumes and Lemma Opening After Low Light Stress

**FIGURE 4:**
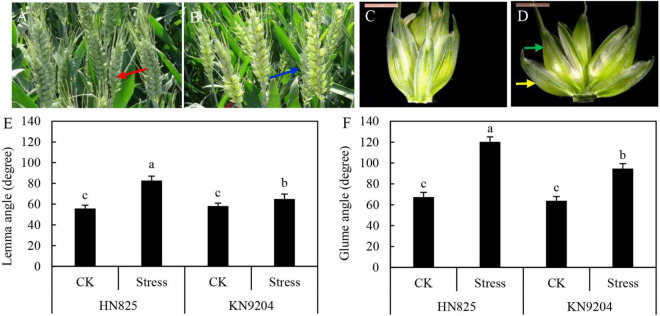
Measurement of glume and lemma angle in ‘HN825’ and ‘KN9204’. Spike **(A,B)** and spikelet **(C,D)** images of control **(A,C)** and low light stress **(B,D)** at early grain filling stage (20 days after low light treatment). **(E)** The lemma angle. **(F)** The glume angle. The red arrows indicate the spike in control **(A)**, the blue arrows indicate the spike in stressed spike **(B)**, the yellow arrows indicate the glume, and the green arrows indicate the lemma **(D)**. Different letters indicate the statistically significant differences between treatments (*P* < 0.05). Scale bars are 5 mm.

To explore the causes why wheat cross-pollination occurred, the morphology and color of stressed spikelets and control spikelets were investigated. And we found the color of stressed spike was lighter than that of the control spike at the early grain filling stage (Figures 4A,B). Moreover, glume and lemma in the stressed spikelet were open, while the control spikelet was tightly closed (Figures 4C,D). Compared to control, the opening degree of lemma and glume were increased by 11.6–48.6, 48.4–78.5%, respectively (Figures 4E,F). Moreover, the opening degree of the glume was larger than that of the lemma. The glume opening in ‘HN825’ was larger than that in ‘KN9204’, which is consistent with the compensation rate. These results suggested that increasing the glume opening might increase the ovary cross-pollination rate.

### Content of Jasmonic Acid, Methyl Jasmonate, Lignin, and Cellulose in Spikelet

**FIGURE 5:**
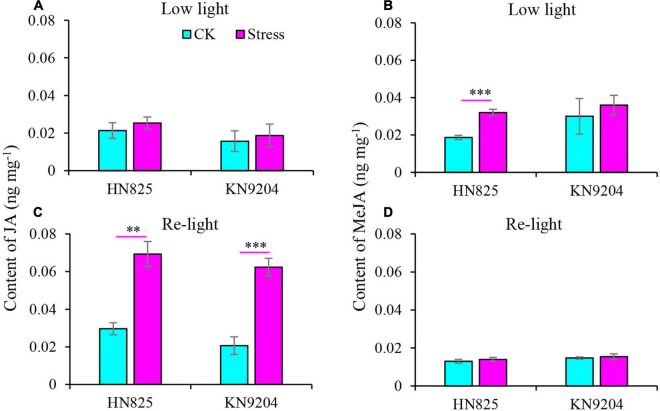
Effect of low light on the contents of jasmonic acid (JA) **(A,C)** and methyl-jasmonate (MeJA) **(B,D)** in the spikelet of two wheat cultivars (‘HN825’ and ‘KN9204’). Low light, under low light stress; Re-light, early grain filling stage (20 days after low light treatment). Significant differences between the CK and stress treatments are indicated by asterisks (**P < 0.01; ***P < 0.001, Student’s *t*-test).

**FIGURE 6:**
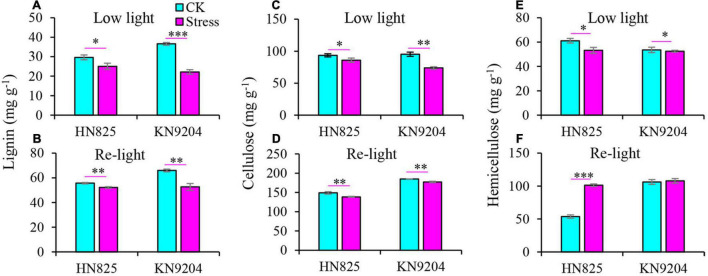
Effect of low light on the contents of lignin **(A,B)**, cellulose **(C,D)**, and hemicellulose **(E,F)** in the spikelet of two wheat cultivars (‘HN825’ and ‘KN9204’). Low light, under low light stress; Re-light, early grain filling stage (20 days after low light treatment). Significant differences between the CK and stress treatments are indicated by asterisks (*P < 0.05; **P < 0.01; ***P < 0.001, Student’s *t*-test).

A previous study reported that JA and MeJA content increased upon the spikelet opening (Chen et al., 2020). Therefore, the JA and MeJA content was measured in a spikelet (Figure 5). Compared to control, the content of JA and MeJA in spike increased under low light stress (Figures 5A,B). Moreover, the content of JA was constantly higher in stressed spikelets compared to control from the point when the stress was removed to the early grain filling stage (Figure 5C). To further investigate the glume and lemma opening, we dissected glume and lemma under low light and at the early grain filling stage (20 days after low light treatment) to measure the content of lignin, cellulose, and hemicellulose. Results showed that the lignin and cellulose of glume from the low light treatment group significantly decreased as compared with CK at the two-time point (Figures 6A–D). Statistically, the lignin and cellulose were reduced by 15.5–39.4 and 8.0–22.5% in the low light treatment group, by 6.3–20.2 and 4.2–7.2% after low light treatment. In addition, hemicellulose of glume only significantly decreased in the low light treatment group, while it was not hampered after low light treatment (Figures 6E,F). This suggested that low light has a negative influence on the lignin and cellulose of glume, increasing the opening of the glume.

### RNA-Seq Finding Key Terms Responsible for Floret Opening

**FIGURE 7:**
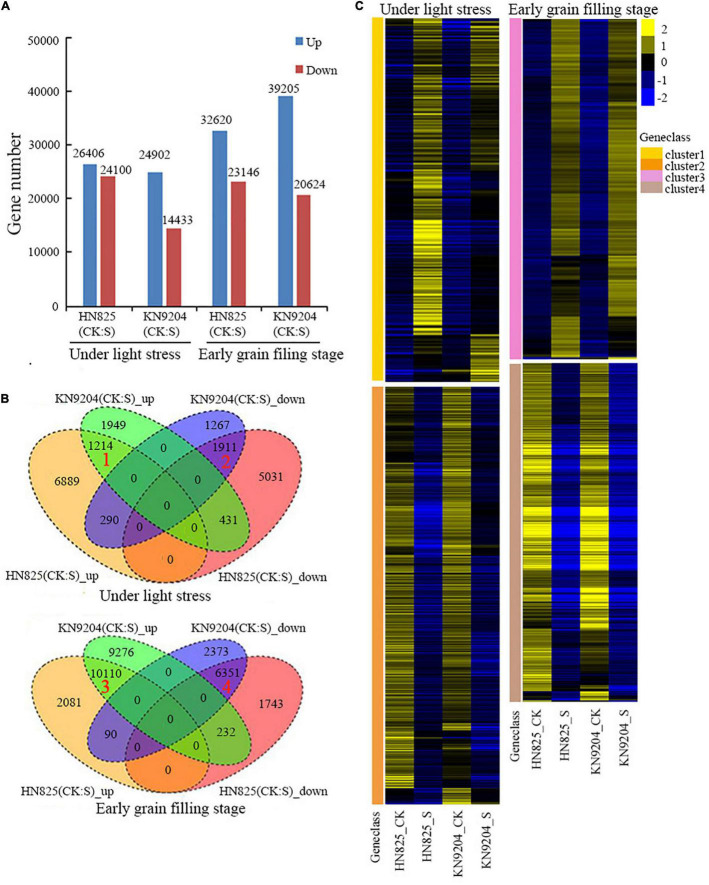
Analysis of RNA-sequencing data of the spike in low light stress (during young microspore) and early grain filling stage (20 days after low light treatment) under low light stress (S) and control (CK) conditions. **(A)** The numbers of the differentially expressed genes (DEGs). **(B)** Venn diagram analysis of all up-regulated and down-regulated DEGs in two wheat cultivars (‘HN825’ and ‘KN9204’). **(C)** Heatmap clustering of the up-regulated (cluster 1 and 3) and down-regulated (cluster 2, 4) in both cultivars under low light stress and early grain filling stage.

To investigate the molecular mechanisms underlying spike fertility and the opening of the glume, RNA-seq analysis was performed using the spike tissues of the ‘HN825’ and ‘KN9204’ cultivars collected on the fifth day of low light stress and at early grain filling stage (20 days after removing low light). The DEGs between CK and stress treatment at the two-time point were identified based on their normalized expression level. The number of the DEGs in each comparison ranged from 14433 to 39205 (Figure 7A and Supplementary Dataset 1). Four clusters of genes were selected for further analysis. Cluster 1 and cluster 2 represented overlapped up-regulated or down-regulated genes under stress on the fifth day of low light stress. Cluster 3 and cluster 4 represented overlapped up-regulated or down-regulated genes under stress on the 20th day after low light treatment (Figures 7B,C).

**FIGURE 8:**
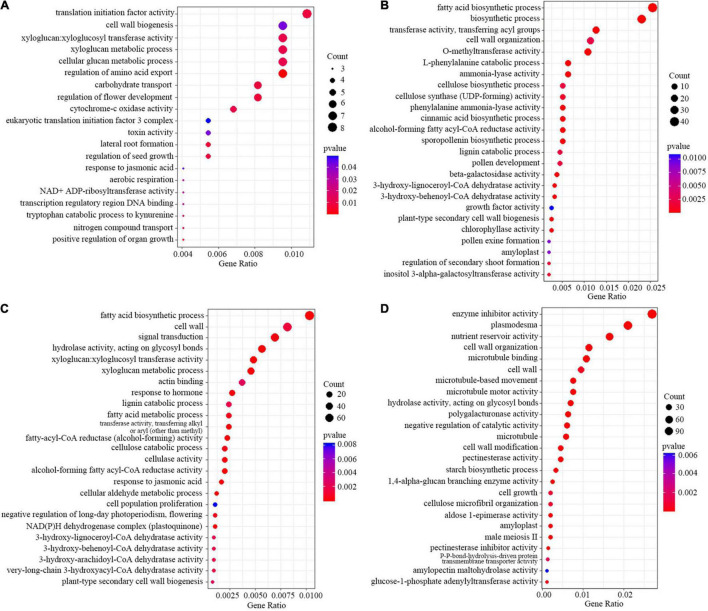
Scatterplots showing the GO enrichment results of cluster 1 **(A)**, 2 **(B)**, 3 **(C)**, and 4 **(D)**.

Gene ontology enrichment analysis found that several nutrients transport related GO terms such as “carbohydrate transport”, “nitrogen compound transport”, and “cytochrome-c oxidase activity”, “regulation of flower development”, and “response to jasmonic acid” enriched for up-regulated DEGs under low light group (Figure 8A and Supplementary Dataset 2). For the down-regulated genes in cluster 2, genes related to fatty acid biosynthesis metabolism pathways, and genes involved in the biosynthesis and inactivation process of the cell wall and lignin and cellulose were also enriched (Figure 8B). In addition, lignin and cellulose catabolic related terms, response to jasmonic acid related terms were enriched in the up-regulated genes in the early grain filling stage (Figure 8C and Supplementary Dataset 3). For the down-regulated genes in the early grain filling stage, GO terms, such as “nutrient reservoir activity”, “starch biosynthetic process”, “pectinesterase activity”, and “pectinesterase inhibitor activity” were enriched (Figure 8D). Moreover, phenylalanine metabolism related KEGG term was enriched in the down-regulated genes under low light group (Supplementary Dataset 4). The results suggested that the lignin and cellulose degradation might play an important role in glume opening under low light conditions, consistent with the content changes of lignin and cellulose in different stages in our previous data.

**FIGURE 9:**
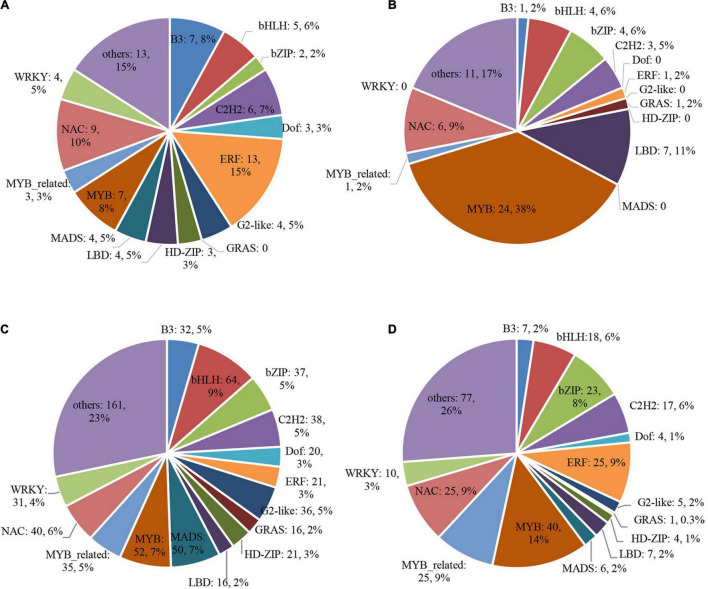
Differentially expressed transcription factor (TF) genes. **(A)** cluster 1; **(B)** cluster 2; **(C)** cluster 3; **(D)** cluster 4.

In addition, we checked all the transcription factors (TFs) in the DEGs of four clusters. There were 88 and 64 TFs in the up- and down-regulated DEGs in low light treatment groups (Figures 9A,B), 710 and 294 TFs in the up- and down-regulated DEGs after low light treatment groups, respectively (Figures 9C,D). Many MYB and LBD family TFs were enriched in the down-regulated DEGs, but only a few were found in the up-regulated DEGs in low light treatment groups. Homology comparison showed that most of the homologous genes of down-regulated TFs in Arabidopsis could modulate xylem development or secondary cell wall biogenesis (Supplementary Dataset 5). This implies that they might participate in the regulation of glume opening process in wheat.

## Discussion

In cereals, flowering is related to the capacity of seed production, which is more sensitive to abiotic stress in booting stage (Ji et al., 2010). Due to climate change, adverse stress seriously threatened flowering and seed setting, eventually resulting in lower yields (Dong et al., 2017). However, plants exhibit various mechanisms for surviving, including morphological adaptions under abiotic stress conditions (Hasanuzzaman et al., 2013). For self-pollination wheat, when the ovary was not pollinated a few days after anthesis, the cross-pollination was carried out by changing its cleistogamic (closed) floral morphologies (Okada et al., 2018), and a similar phenomenon after low light stress was found in our study. Low light stress reduces the fertility of anther and ovaries, while the ovaries are less affected and still fertile. To receive fertile pollens for florets with normal ovaries but sterile anthers, wheat adopts the strategy of capturing heterologous pollen to finish pollination. In the present study, grain number per spike significantly decreased by 58.5–98.5% due to unsuccessful self-pollination after low light stress. However, the compensation ratio of grain number per spike due to accepting heterologous pollen grain was 2.1–18.0%. The compensatory capacity of ‘KN9204’ was lower than that of ‘HN825’, probably because the spike layer of ‘KN9204’ variety was neatly grown and flowered uniformly, which made it difficult for the ovary to capture heterologous mature pollen. Taken together, the change of wheat pollination ways after low light stress encountered at the YM stage could serve as a compensation strategy to alleviate the reduction of yield, which is of great significance for ensuring food security under adverse climate conditions.

For the male sterile florets in wheat, the separation angle of the glumes and lemma is important for ensuring the exsertion of stigmas (Chen et al., 2020). In present study, the glume and lemma opened greatly after low light removal till the early grain filling stage. The opening angles of lemma and glume were increased by 11.6–48.6, 48.4–78.5% compared to control, respectively. This ensures that the ovaries can receive heterologous fertile pollens brought by the wind. Glume and lemma opening is a trait that is related not only to spikelet position within the spike, spikelet density, and ovary size but also to the glume stiffness (Okada et al., 2018). The main factors influencing glume toughness are cellulose and lignin metabolism, and cell wall metabolism (Zou et al., 2015). In this study, we found that several biological processes related to carbohydrate metabolism, response to cell wall macromolecular metabolism were involved in glume opened spikes after low light stress. Both down-regulated DEGs were significantly enriched in the cell wall organization process and up-regulated DEGs were significantly enriched in lignin metabolism, fatty acid metabolic process, and response to the jasmonic acid process. These results indicate that cell wall metabolism and the jasmonic acid process may play a key role in the opening of wheat glumes after low light stress.

Plant growth and development are modulated by complex molecular networks in which transcription factors (TFs) played a central role in regulating other gene expressions (Ramachandran et al., 1994; Riechmann et al., 2000; Mitsuda and Ohme-Takagi, 2009). We identified TFs with altered expression in our set of DEGs with common expression patterns in both cultivars. Most of the homologous genes in Arabidopsis of these TFs (such as MYB, LBD, ERF NAC, and MADS family TFs) have functions related to the cell wall biogenesis. They may be involved in regulating glume opening. Meanwhile, lignin and cellulose contents in spikelets were significantly reduced compared with the control. This further verified that lignin and cellulose might be the main factors leading to glume opening.

Phytohormones have been considered the main factor regulating the spikelet opening. JA and MeJA can promote the opening of crops glumes (Zeng et al., 1999; Liu et al., 2017; Yang J. et al., 2020). The glume opening and increased level of JA and MeJA were also found after low light stress, strongly demonstrating that JA and MeJA might play an important role in the glume opening and capturing heterogenous pollens after stresses. Thus, the application of JA or MeJA may be a useful method to guarantee grain number and grain yield when wheat encounter stresses which can induce male sterility.

## Conclusion

Compared with female organs, anther was more sensitive to environmental stimulation, such as low light stress. When the stress was imposed during the microspore stage, the male is partially or completely sterile. Male sterility leads to failure of self-pollination, which leads to serious yield loss. However, the active ovary can compensate for the loss of yield caused by pollen abortion by receiving active pollen from different sources. In the process of glume and lemma opening, the cellulose and lignin decrease induced by down-regulated key metabolism- or signaling-related genes played an important role, which is necessary for cross-pollination. Cross-pollination of wheat provides a survival mechanism to set seeds in the absence of self-fertilization after the stress of adversity. Thus, pollination way alteration may be a method for plants to adjust to a stressful environment and can be used in agriculture production to guarantee a stable yield under extreme weather.

## Data Availability Statement

The datasets presented in this study can be found in online repositories. The names of the repository/repositories and accession number(s) can be found below: https://www.ncbi.nlm.nih.gov/sra; PRJNA835521.

## Author Contributions

BD, HY, and ML designed the study. HY, YQ, WL, YM, and WQ performed the experiments. HY and YL contributed data analysis. HY prepared the manuscript. BD, CL, HS, LL, and DL supervised this project and the manuscript. All authors contributed to the article and approved the submitted version.

## Conflict of Interest

The authors declare that the research was conducted in the absence of any commercial or financial relationships that could be construed as a potential conflict of interest.

## Publisher’s Note

All claims expressed in this article are solely those of the authors and do not necessarily represent those of their affiliated organizations, or those of the publisher, the editors and the reviewers. Any product that may be evaluated in this article, or claim that may be made by its manufacturer, is not guaranteed or endorsed by the publisher.

## References

[B1] Cardona O. van Aalst M. Birkmann J. M. Fordham M. McGregor G. Perez R. (2012). *Determinants of Risk: Exposure and Vulnerability, in Managing the Risks of Extreme Events and Disasters to Advance Climate Change Adaptation. A Special Report of Working Groups I and II of the Intergovernmental Panel on Climate Change (IPCC).* Cambridge, MA: Cambridge University Press, 65–108.

[B2] Chameides W. L. Yu H. Liu S. C. Bergin M. Zhou X. Mearns L. (1999). Case study of the effects of atmospheric aerosols and regional haze on agriculture: an opportunity to enhance crop yields in China through emission controls? *Proc. Natl. Acad. Sci. U.S.A.* 96 13626–13633. 10.1073/pnas.96.24.13626 10570123 PMC24115

[B3] Chen C. Baethgen W. E. Robertson A. (2013). Contributions of individual variation in temperature, solar radiation and precipitation to crop yield in the North China Plain, 1961–2003. *Clim. Change* 116 767–788. 10.1007/s10584-012-0509-2

[B4] Chen J. Xu Y. Fei K. Wang R. Yang J. (2020). Physiological mechanism underlying the effect of high temperature during anthesis on spikelet-opening of photo-thermo-sensitive genic male sterile rice lines. *Sci. Rep.* 10:2210. 10.1038/s41598-020-59183-0 32042005 PMC7010791

[B5] Christiaens A. De Keyser E. Pauwels E. De Riek J. Gobi B. Van Labek M. C. (2016). Suboptimal light conditions influence source-sink metabolism during flowering. *Front. Plant Sci.* 7:249. 10.3389/fpls.2016.00249 26973689 PMC4776122

[B6] de Vries A. (1971). Flowering biology of wheat, particularly in view of hybrid seed production—a review. *Euphytica* 20 152–170. 10.1007/BF00056076

[B7] Dong B. D. Yang H. Liu H. P. Qiao Y. Z. Zhang M. M. Wang Y. K. (2019). Effects of shading stress on grain number, yield, and photosynthesis during early reproductive growth in wheat. *Crop Sci.* 59 363–378. 10.2135/cropsci2018.06.0396

[B8] Dong B. D. Zheng X. Liu H. P. Able J. A. Yang H. Zhao H. (2017). Effects of drought stress on pollen sterility, grain yield, abscisic acid and protective enzymes in two winter wheat cultivars. *Front. Plant Sci.* 8:1008. 10.3389/fpls.2017.01008 28676806 PMC5476748

[B9] Endo M. Tsuchiya T. Hamada K. Kawamura S. Yano K. Ohshima M. (2009). High temperatures cause male sterility in rice plants with transcriptional alterations during pollen development. *Plant Cell Physiol.* 50 1911–1922. 10.1093/pcp/pcp135 19808807

[B10] Fischer R. A. (2011). Wheat physiology: a review of recent developments. *Crop Pasture Sci.* 62 95–114. 10.1071/CP10344

[B11] Food and Agriculture Organization [FAO] (2013). *FAO Statistical Yearbook.* Rome: FAO.

[B12] Foulkes M. J. Slafer G. A. Davies W. J. Berry P. M. Sylvester-Bradley R. Martre P. (2011). Raising yield potential of wheat. III. Optimizing partitioning to grain while maintaining lodging resistance. *J. Exp. Bot.* 62 469–486. 20952627 10.1093/jxb/erq300

[B13] Fu C. Wang F. Liu W. Liu D. Li J. Zhu M. (2017). Transcriptomic analysis reveals new insights into high-temperature-dependent glume-unclosing in an elite rice male sterile line. *Front. Plant Sci.* 8:112. 10.3389/fpls.2017.00112 28261226 PMC5306291

[B14] Griffin W. B. (1987). Outcrossing in New Zealand wheats measured by occurrence of purple grain. *New Zeal. J. Agric. Res.* 30 287–290. 10.1080/00288233.1987.10421885

[B15] Hasanuzzaman M. Nahar K. Alam M. Roychowdhury R. Fujita M. (2013). Physiological, biochemical, and molecular mechanisms of heat stress tolerance in plants. *Int. J. Mol. Sci.* 14 9643–9684. 10.3390/ijms14059643 23644891 PMC3676804

[B16] Heslop-Harrison Y. Heslop-Harrison J. S. (1996). Lodicule function and filament extension in the grasses: potassium ion movement and tissue specialization. *Ann. Bot.* 77 573–582. 10.1093/aob/77.6.573

[B17] Hucl P. (1996). Out-crossing rates for 10 Canadian spring wheat cultivars. *Can. J. Plant Sci.* 76 423–427. 10.4141/cjps96-075

[B18] Jain M. Prasad P. V. Boote K. J. Jr. Hartwell A. L. Chourey P. S. (2007). Effects of season-long high temperature growth conditions on sugar-to-starch metabolism in developing microspores of grain sorghum (*Sorghum bicolor* L. Moench). *Planta* 227 67–79. 10.1007/s00425-007-0595-y 17680267

[B19] Ji X. Shiran B. Wan J. Lewis D. C. Jenkins C. L. Condon A. G. (2010). Importance of preanthesis anther sink strength for maintenance of grain number during reproductive stage water stress in wheat. *Plant Cell Environ.* 33 926–942. 10.1111/j.1365-3040.2010.02130.x 20199626

[B20] Ji X. M. Dong B. D. Shiran B. Talbotet M. J. Edlington J. E. Hughes T. (2011). Control of abscisic acid catabolism and abscisic acid homeostasis is important for reproductive stage stress tolerance in cereals. *Plant Physiol.* 156 647–662. 10.1104/pp.111.176164 21502188 PMC3177265

[B21] Kirby E. J. M. Fellowes G. Appleyard M. (eds) (1983). “Anthesis in winter barley,” in *Annual Report—Plant Breeding Institute*, (Cambridge, MA: Cambridge Plant Breeding Institute), 112–113.

[B22] Li Y. Fu X. Zhao M. Zhang W. Li B. An D. (2018). A genome-wide view of transcriptome dynamics during early spike development in bread wheat. *Sci. Rep.* 8:15338. 10.1038/s41598-018-33718-y 30337587 PMC6194122

[B23] Liu L. I. Zou Z. Qian K. E. Xia C. He Y. Zeng H. (2017). Jasmonic acid deficiency leads to scattered floret opening time in cytoplasmic male sterile rice Zhenshan 97A. *J. Exp. Bot.* 68 4613–4625. 10.1093/jxb/erx251 28981770 PMC5853226

[B24] Llorens C. Argentina M. Rojas N. Westbrook J. Dumais J. Noblin X. (2016). The fern cavitation catapult: mechanism and design principles. *J. R. Soc. Interface* 13:20150930. 10.1098/rsif.2015.0930 26763327 PMC4759797

[B25] Martin T. J. (1990). Outcrossing in 12 hard red winter-wheat cultivars. *Crop Sci.* 30 59–62. 10.2135/cropsci1990.0011183X003000010013x

[B26] Mitsuda N. Ohme-Takagi M. (2009). Functional analysis of transcription factors in *Arabidopsis*. *Plant Cell Physiol.* 50 1232–1248. 10.1093/pcp/pcp075 19478073 PMC2709548

[B27] Nguyen V. Fleury D. Timmins A. Laga H. Hayden M. Mather D. (2015). Addition of rye chromosome 4R to wheat increases anther length and pollen grain number. *Theor. Appl. Genet.* 128 953–964. 10.1007/s00122-015-2482-4 25716820

[B28] Okada T. Ridma J. E. A. Jayasinghe M. Nansamba M. Baes M. Warner P. (2018). Unfertilized ovary pushes wheat flower open for cross-pollination. *J. Exp. Bot.* 69 399–412. 10.1093/jxb/erx410 29202197 PMC5853862

[B29] Ostergaard L. (2009). *Fruit Development and Seed Dispersal (L. Ostergaard, Ed.).* Oxford: Wiley.

[B30] Pickett A. (1993). *Hybrid Wheat Results and Problems.* Berlin: Paul Parey Scientific Publishers.

[B31] Ramachandran S. Hiratsuka K. Chua N. H. (1994). Transcription factors in plant growth and development. *Curr. Opin. Genet. Dev.* 4 642–646. 10.1016/0959-437X(94)90129-Q 7849502

[B32] Riechmann J. L. Heard J. Martin G. Reuber L. Jiang C. Keddie J. (2000). *Arabidopsis* transcription factors: genome-wide comparative analysis among eukaryotes. *Science* 290 2105–2110. 10.1126/science.290.5499.2105 11118137

[B33] Solomon S. D. Qin D. Manning M. Chen Z. Miller H. L. (2007). *Climate Change 2007: The Physical Science Basis. Working Group I Contribution to the Fourth Assessment Report of the IPCC.* Cambridge, MA: Cambridge University Press.

[B34] Wang L. Deng F. Ren W. J. (2015). Shading tolerance in rice is related to better light harvesting and use efficiency and grain filling rate during grain filling period. *Field Crops Res.* 180 54–62.

[B35] Wang Z. Gu Y. J. (2012). The process of opening and closing of florets in rice and its influencing factors. *Sci. Pap.* [Epub ahead of print].

[B36] Wang Z. Lu C. M. Gu Y. J. Gao Y. Z. (1988). Studies on the mechanism of the anthesis of rice I. Effect of temperature on spikelet opening and pollen vigor. *Acta Agron. Sin.* 14 14–21.

[B37] Xie G. S. Zeng H. L. Lin X. H. Tao A. L. Zhang D. P. (2003). Effect of temperature on the fertility restoration of different two-line hybrid rice. *J. Genet. Genomics* 30 142–146. 12776602

[B38] Yang H. Dong B. D. Wang Y. K. Qiao Y. Z. Shi C. H. Jin L. L. (2020). Photosynthetic base of reduced grain yield by shading stress during the early reproductive stage of two wheat cultivars. *Sci. Rep.* 10:14353. 10.1038/s41598-020-71268-4 32873861 PMC7463241

[B39] Yang J. Fei K. Chen J. Wang Z. Zhang W. Zhang J. (2020). Jasmonates alleviate spikelet-opening impairment caused by high temperature stress during anthesis of photo-thermo-sensitive genic male sterile rice lines. *Food Energy Secur.* 6:e233. 10.1002/fes3.233

[B40] Zajczkowska U. Denisow B. Lotocka B. Dolkin-Lewko A. Rakoczy-Trojanowska M. (2021). Spikelet movements, anther extrusion and pollen production in wheat cultivars with contrasting tendencies to cleistogamy. *BMC Plant Biol.* 21:136. 10.1186/s12870-021-02917-7 33726675 PMC7970976

[B41] Zeng X. C. Zhou X. Zhang W. Murofushi N. Kitahara T. Kamuro Y. (1999). Opening of rice floret in rapid response to methyl jasmonate. *J. Plant Growth Regul.* 18 153–158. 10.1007/PL00007063 10688703

[B42] Zou H. Tzarfati R. Hubner S. Krugman T. Fahima T. Abbo S. (2015). Transcriptome profiling of wheat glumes in wild emmer, hulled landraces and modern cultivars. *BMC Genom.* 16:777. 10.1186/s12864-015-1996-0 26462652 PMC4603339

